# Spectral Filter Selection for Increasing Chromatic Diversity in CVD Subjects

**DOI:** 10.3390/s20072023

**Published:** 2020-04-03

**Authors:** Miguel Ángel Martínez-Domingo, Eva M. Valero, Luis Gómez-Robledo, Rafael Huertas, Javier Hernández-Andrés

**Affiliations:** Department of Optics, University of Granada, 18071 Granada, Spain; martinezm@ugr.es (M.Á.M.-D.); luisgrobledo@ugr.es (L.G.-R.); rhuertas@ugr.es (R.H.); javierha@ugr.es (J.H.-A.)

**Keywords:** aids for the visually impaired, spectral imaging, color imaging, color blindness

## Abstract

This paper analyzes, through computational simulations, which spectral filters increase the number of discernible colors (NODC) of subjects with normal color vision, as well as red–green anomalous trichromats and dichromats. The filters are selected from a set of filters in which we have modeled spectral transmittances. With the selected filters we have carried out simulations performed using the spectral reflectances captured either by a hyperspectral camera or by a spectrometer. We have also studied the effects of these filters on color coordinates. Finally, we have simulated the results of two widely used color blindness tests: Ishihara and Farnsworth–Munsell 100 Hue (FM100). In these analyses the selected filters are compared with the commercial filters from EnChroma and VINO companies. The results show that the increase in NODC with the selected filters is not relevant. The simulation results show that none of these chosen filters help color vision deficiency (CVD) subjects to pass the set of color blindness tests studied. These results obtained using standard colorimetry support the hypothesis that the use of color filters does not cause CVDs to have a perception similar to that of a normal observer.

## 1. Introduction

The study of color vision deficiencies (CVDs) has recently gained attention from social networks due to the appearance of companies that have developed what they call "aids" for subjects with CVD. Approximately 1 in 12 men and 1 in 200 women have some type of CVD [1]. These deficiencies are usually classified according to the type of cone affected and the severity of the CVD. Normal color vision observers have three different kinds of light photoreceptors in their retinas. These three cones are called L, M, and S because they roughly respond to long, medium, and short wavelength ranges of the visible spectrum. The most frequent anomalies are those of the red–green type [2], which, according to the criteria mentioned, can be divided into two types: protan and deutan, depending on the cone that is affected (L or M, respectively). If the affected cone is S, a blue–yellow anomaly occurs: tritan. In the case of subjects having the three types of cones (L, M, and S), but if one of them is anomalous, they are called anomalous trichromats (protanomalous, deuteranomalous, or tritanomalous depending on whether the affected cone is L, M, or S respectively), and if they totally lack any type of cone then they are called dichromats (protanopes, deuteranopes, or tritanopes depending on whether they lack the L, M, or S cone, respectively). In this study we have not taken into account tritanomalous subjects or tritanopes, since these represent a low percentage of the CVD population (according to [3]).

Subjects whose color vision anomaly is between moderate and severe may suffer limitations in their daily lives, such as not distinguishing the degree of cooking of meat, the maturity of certain fruits and vegetables, the color of certain indicative lights (LEDs), different colors on maps, etc. For this reason, in most countries, these subjects are automatically excluded from certain jobs such as being an airplane pilot, firefighter, train driver, air traffic controller, etc. [4]. 

Although genetic therapy experiments in mice and primates have been carried out [5], there are still no effective treatments in humans to reverse this condition [2]. The fields of engineering and colorimetry have paid special interest to the study of anomalous color vision. Both have worked hand in hand to develop active tools that work by image processing to help these subjects in their daily tasks [6,7]. However, this type of aid, although useful for performing certain tasks, requires the use of displays to artificially increase the contrast perceived by CVD subjects, and depends on the specific task to be facilitated.

On the other hand, passive aid is of interest, such as filters that can be worn in glasses or contact lenses. Despite the lack of scientific evidence on the improvement of color vision in CVD observers using any type of filters, companies manufacturing them (such as EnChroma Inc. [8], founded in 2010, or VINO Optics [9], founded in 2006, amongst others) are gaining attention with commercial campaigns advertising improvement on the color vision of CVD observers wearing their products.

This viral phenomenon does not go unnoticed by many researchers who study the effect of this type of passive aid from different paradigms [10,11,12,13,14]. Real CVD observers have been used in all this research to study how their color vision changes when the passive aids are used, and if it becomes more similar to that of subjects with normal color vision. None of these studies have concluded that the use of these aids allows CVD observers to achieve a vision similar to normal observers.

In addition, some authors have carried out research using computational calculations in a complementary way to simulate the different conditions of CVD [10,11,15,16,17]. Working with simulations is a very interesting and powerful complement, specifically if you want to determine if a filter is effective or not, since it allows you to study a large number of filters and reflectances representative of a very varied set of real objects, as well as different CVD conditions. In addition, you can study the effect of each of the filters on different aspects of color vision (how they affect each of the perceptual attributes separately, distributions in the color space, simulate the effect on the result of different tests of color vision, etc.). It is in this aspect that the methods of capturing and processing spectral images become very important. This type of technology allows us to take into account the effect of small modulations on the spectral transmittance of filters for the precise calculation of color coordinates, thus opening up the possibility of using stimuli in a realistic context such as real natural or urban scenes.

In this study, we have used computer simulations using reflectance data measured with spectral capture systems. With this data we have verified the effectiveness of the use of spectral filters to increase the number of discernible colors (NODC) for different types of observers, as well as the effect that these filters have on color coordinates. At this point we would like to clarify that, as defined in Section 2.3.2, NODC is related to chromatic diversity (as explained in [18]) without being considered an exact measure of the actual number of colors that a subject could discern.

Other authors have done work in this regard. Linhares et al. [18] investigated computationally the effects of only 10 colored lenses on the NODC perceived by dichromats, using the algorithm of Brettel et al. [19] to model dichromatic vision and to simulate for normal observers the appearance of 50 hyperspectral images (with a spectral resolution of 10 nm) of natural scenes for the three types of dichromats (protanopes, deuteranopes, and tritanopes). They found that in dichromatic vision the NODC was around 7% of that perceived by normal trichromatic observers. Nine of the lenses tested were commercial sunglasses with colored lenses. The other lens was an optimized lens that maximizes the NODC perceived by normal trichromats when seeing the natural scenes. The effect of these lenses on normal trichromats was a small enhancement on the NODC. However, for protanopes and deuteranopes the effect was a small impairment, whereas for tritanopes there was a considerable enhancement. The authors also claimed that whether these improvements could be perceived by observers as enhancing their color vision is still an open question [18].

Moreland et al. studied the effect of filters by simulations in [20]. In this case, instead of standard colorimetry, they worked in LMS cone responses space using the cone sensitivity functions proposed in [21]. They calculated the standard deviation along some cone responses ratios, and defined specific enhancement factors with them. They studied 43 commercial filters and the reflectances from 658 Munsell samples and three standard traffic signals colors under C illuminant. This procedure is interesting for quantifying the effect of the filters, but it does not allow study of the changes in the perceptual attributes, or the impact on CVD tests like we do in this work

Marín Franch and Foster [22] used information–theoretic methods to estimate NODC taking into account both differing surface-color frequencies and observer response uncertainty. However, they neither simulated CVD observers nor the effect of filters. They obtained much smaller values than those based on counting methods, such as the one used in this study.

Pastilha et al. [23] evaluated the NODC through a psychophysical experiment to test the hypothesis that pairs of colors confused by dichromats are not frequent in natural scenes and therefore the visual impairment in natural environments is not as high as predicted by other studies [18]. Four normal trichromats and four dichromats (two protanopes and two deuteranopes) participated in the experiment and the results showed that the number of pairs discriminated by dichromats was around 70% of those discriminated by normal trichromats.

Masuda and Nascimento [24] studied different illuminant spectra in order to maximize the colorfulness of the set of optimal colors proposed by Schrödinger [25]. This problem was similar to a filter optimization problem. However, instead of the NODC, the evaluation metric they used to select the optimal illuminant was the volume of the solid generated by the Schrödinger’s optimal colors under the different illuminants, which is related to their discrimination capabilities. This idea was already proposed by Thorton [26]. Masuda and Nascimento conclude with the idea that the effect of selecting such an illuminant would be similar to using colored filters with absorption bands at certain wavelengths (notch filters like the ones studied in this work).

In this study, we have investigated three types of filters: band-pass and notch whose transmittances have been simulated, and the two commercial models studied in previous research, from EnChroma [11] and VINO [10] companies. Differently from the works cited above, we have considered a huge set of 90,922 filters (see 2.1.c). We have evaluated how the filters affect NODC in different sets of objects and scenes of diverse origin, unlike the cited previous works. In addition, both dichromatic subjects and anomalous trichromatic subjects and subjects with normal color vision have been simulated. Also, the effects of each filter for each type of observer on the result of the Ishihara test [27] and, for the first time, the FM100 [28] test have been simulated. As far as we know, such a large set of filters has never been studied, nor have so many different CVD conditions been covered.

## 2. Methods

### 2.1. Reflectances

In this study the spectral information was limited within the range from 400 nm to 700 nm (visible range), and sampled every 1 nm through linear interpolation. Three reflectance sets were used, each of them corresponding to three different types of objects or scenes. Simulations were carried out using D65 as illuminant following CIE (Commission Internationale de l’Eclairage) recommendations for the use of CIELAB color space [29]. This is a representation of daylight with a correlated color temperature of approximately 6500 K.

#### 2.1.1. Data Set 1 (D1: Atlas)

**Figure 1:**
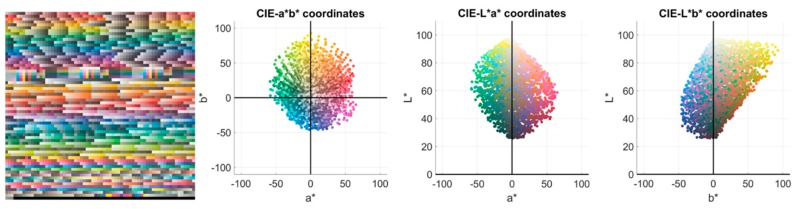
sRGB (i.e. the standard RGB space [33]) rendering of the 4,233 samples present in D1 under illuminant D65, and their L*a*b* projections.

In order to select which filters, amongst the 90,922 simulated, maximized the NODC for each observer, a reduced but diverse hyperspectral reflectance set was used. This set was composed of the reflectances present in four popular color atlases: Munsell (1269 samples) [30], NCS (1751 samples) [31], Agfa (290 samples), and Pantone (923 samples) [32]. This set was used because it covers the whole color space quite regularly. Moreover, comparisons with other studies were easier this way. There were 4,233 samples in total in D1. Figure 1 shows an sRGB (i.e. the standard RGB space) [33] rendering of the samples, as well as the three bi-dimensional projections of their L*, a*, b* distribution in CIELAB color space.

#### 2.1.2. Data Set 2 (D2: Scenes)

**Figure 2:**
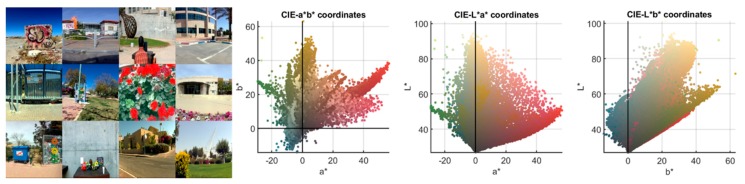
sRGB rendering (i.e. into the standard RGB space [33]) of the 12 scenes present in D2 (selected from a hyperspectral image database [30]) under illuminant D65, and their L*a*b* projections.

This set is composed of 12 urban scenes retrieved from a database of hyperspectral images [34]. These scenes were chosen because of their color diversity, as well as for being representative of outdoor urban scenes for the majority of the population. These images were originally captured within the range from 400 to 1000 nm, with a step of 1.25 nm (519 bands). They were then interpolated to the previously mentioned visible range between 400 and 700 nm with 1 nm steps (301 bands). Figure 2 (left) shows the sRGB rendering [33] of the chosen scenes.

The size of each spectral image was 1,300 x 1,392 pixels, which means a total of 21,715,200 spectral reflectances in D2. As mentioned in Section 1, the total number of filters simulated was 90,922. Since the amount of data is huge, the simulations would be computationally very slow (more than three months using 16 GB RAM and 3.19 GHz CPU). Hence, this set has been used to study the effect on the color coordinates only of the filters selected using D1. Figure 2 (right) shows the L*, a*, b* distribution of D2 under D65 illuminant on its three planar projections. Compared with D1 (Figure 1), one can see how, even though D1 contains less reflectances, its chromatic diversity is wider since it covers a larger gamut compared to that typically found in natural scenes.

#### 2.1.3. Data Set 3 (D3: Ishihara and FM100)

The third data set was composed of samples from two CVD tests. The first one was the Ishihara color vision anomaly detection test [27,35]. Six plates were chosen (pages 2, 3, 6, 7, 22, and 23) to be representative of this test. Their reflectances were measured using a hyperspectral scanner model Resonon Pika L [10,36]. This hyperspectral scanner together with its linear scanning stage, yielded, after calibration with a reference white and black image, a spectral reflectance image within the range from 383 nm to 1016 nm, with a 4.1 nm step (thanks to the hardware binning technique). After interpolation, the final reflectances were in the same range from 400 to 700 nm with a 1 nm step. These images have been used only for testing the visual effect of the filters.

The second test was FM100 [28]. This is a sorting test consisting of 85 chips of similar luminance and chroma, but a different hue. Their reflectances were measured using the Konica Minolta CS2000 spectro-radiometer. These measurements were used to simulate whether the chosen filters can help CVD observers improve their performance scores in this test.

### 2.2. Filters

Two types of filters were simulated (band-pass and notch), and each of them single and double. These types were considered since the existing passive aids usually feature this spectral shape. The filters were simulated using Gaussian functions (for the band-pass ones), and their complementary functions (for the notch ones). In the following sub-sections, we explain what each of these types of filters is like. Since increasing the width of a Gaussian function involves reducing the slope of its flanks, the rise and descent flank of a 10 nm bandwidth (FWHM) Gaussian function was used. These rise and descent flanks are separated more or less depending on the simulated band-pass or notch width of the filters. Thus, the simulated transmittances were more selective than normal width Gaussian functions but their spectral shapes are smoother than the step function, and hence more realistic. The pass-band for each filter (range between a rise flank and a descent flank) was considered with a maximum transmittance of 1. 

#### 2.2.1. Band-Pass Filters

**Figure 3:**
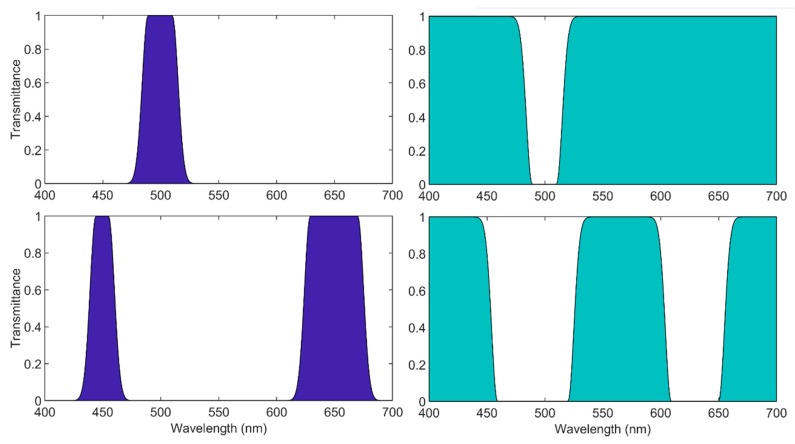
Top: left: spectral transmittances of single band-pass filter. Right: spectral transmittances of single notch filter. Both centered on 500 nm with 30 nm FWHM. Bottom: Left: example of a double band-pass filter. Right: example of double-notch filter.

These filters block all wavelengths but those belonging to a specific spectral range (the so called pass-band). We simulated filters with central wavelengths varying from 400 nm to 700 nm, with a 10 nm step. Moreover, for each central wavelength, we simulated filters where the FWHM bandwidth varies from 10 to 100 nm in 10 steps. This was a total of 310 band-pass filters (see example in Figure 3).

#### 2.2.2. Notch Filters

These filters allow through all wavelengths but those belonging to a specific spectral range (the non-channel or valley). In order to simulate them, all transmittances from the band-pass filters previously calculated were subtracted from the unity transmittance. Thus, another 310 notch filters were simulated (see example in Figure 3).

#### 2.2.3. Double Filters

Along with the single filters, double filters have also been simulated with two pass-bands or two notch-bands. For this purpose, all the possible 2 by 2 band-pass and notch combinations have been simulated (see examples in Figure 3). We get a total of 45,150 double band-pass filters and their complementary 45,150 double-notch filters. This means a total of 90,300 double filters.

#### 2.2.4. Commercial Filters

Together with the previous ones, the transmittances measured from two commercial filters used in previous studies were also included. The models were EnChroma Cx-65 and VINO 02 Amp Oxy-Iso [10,11].

### 2.3. Simulation

The initial data for simulations were: the spectral reflectance *R_object_(λ)*, which multiplied by the spectral radiance of the illuminant *SPD_illum_(λ)*, and by the spectral transmittance of the filter *T(λ)*, yields the color signal *L_color_(λ)*, as shown in Equation (1).

(1) $$L_{color}\left(\lambda \right)=SPD_{illum}\left(\lambda \right)\cdot R_{object}\left(\lambda \right)\cdot T\left(\lambda \right)$$

As mentioned in Section 2.1, the illuminant used was the CIE standard D65, representative of daylight since it is the most common general case of outdoor illumination. From the color signal, and using the CIE1931 Standard Observer, tristimulus values were calculated as well as the color coordinates in CIELAB color space: L*, a*, b*, C*_ab_ y *h_ab_*. The reference white used for both unfiltered and filtered conditions was the same in order to be able to directly compare results between unfiltered and filtered cases.

We need to clarify here that the most correct way to proceed would be to build a personalized set of transformations and functions to be used in colorimetry for each observer (matrix transformations, color space, color difference formulae, perceptual attributes computations, etc.) using as a starting point the color-matching functions of each type of observer. However, this approach, besides being considerably complex, is still an open problem and there is no clear way to see it done in practical terms. Hence, standard colorimetry was used for CVD observers as it is usually done in the literature [10,11,12,14,18], so that results could be compared between different types of observers and simulations could be computed as shown in Section 3.2.2 and Section 3.2.3.

#### 2.3.1. CVD Simulation Model

**Figure 4:**
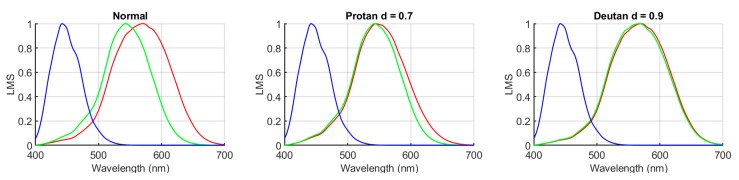
LMS cone response functions simulated for normal (**left**), protanomalous *d* = 0.7 (**center**), and deuteranomalous *d* = 0.9 (**right**) observers.

Once the tristimulus values were calculated for the D1 set and the normal observer, the tristimulus values for different CVD observers (type and severity) were simulated. There are several models in the literature for doing this [15,16,17,19]. In this study, a model proposed by Lucassen and Alferdinck [15] was used. This model has already been explained and used in previous studies by this research group [10,11]. According to this model, the anomalous cone responses for protan (*L’*) and deutan (*M’*) observers are calculated via the following Equations (2) and (3):

(2) $$L^{\prime }=\left(\left(1-d\right)\cdot L\right)+\left(d\cdot M\right)$$

(3) $$M^{\prime }=\left(\left(1-d\right)\cdot M\right)+\left(d\cdot L\right)$$

where *L* and *M* are the cone responses from a normal trichromat observer and parameter *d* represents the amount in which the responses from normal cones are combined to obtain the response from the anomalous cone. Thus, *d* is not directly related to the severity regarded as the spectral separation between *L* and *M* curves. However, this spectral separation is related to the increase of the chromatic discrimination threshold, as shown in [37], where they conclude that a spectral separation of 3 nm between *L* and *M* maxima corresponds to a moderate/severe CVD condition. For this reason, we calculated the value of *d* corresponding to this spectral separation of 3 nm (*d* = 0.7 for protanomalous and *d* = 0.9 for deuteranomalous observers), using the cone response curves in [38]. Figure 4 shows the spectral cone response functions for a normal observer (left), a protanomalous observer (center), and a deuteranomalous observer (right) with the above-mentioned values of *d*.

To simulate dichromat subjects in an extreme case we use the severity parameter with a value of *d* = 1. In this study we have simulated five types of observers: observers with normal color vision, anomalous trichromat observers (protan07 and deutan09), and anomalous dichromat observers (protan10 and deutan10).

#### 2.3.2. Calculation of the Number of Discernible Colors (NODC)

After calculating *XYZ* tristimulus values for the D1 samples and the five different observers simulated both with and without filters, we needed a metric evaluation in order to compare the performance of each filter for each observer. To do so we considered the number of discernible colors (NODC).

If we assume that two colors are discernible when their CIELAB space color difference is above 1 unit (Δ*E_ab_* > 1) [39], we can divide the three-dimensional L*, a*, b* color space in cubes of 1 unit sides, and count in how many of them there is at least one sample [18]. This method has limitations. One is the fact that this color space is not homogeneous; hence the size of the discrimination ellipses is not the same in all the regions. Although more advanced methods have been proposed to calculate the real NODC in a scene [22], this is still an open problem. The aim of this study is not to propose a method to calculate the NODC, but to use this parameter as a measurement of the effect of filters on the CIELAB color distribution. Our aim, rather than knowing the exact number of discernible colors in a scene for each observer, was to determine how this parameter varies for different filters.

## 3. Results

This section is divided into two sub-sections. In the first, the NODC for all the simulated filters is computed, and the filter that maximizes NODC for database D1 is determined. In the second sub-section, the effect of the filters that maximize NODC on color coordinates, sRGB rendering of the scenes, and the Ishihara and FM-100 simulations are explained.

### 3.1. Selection of Filters

**Figure 5:**
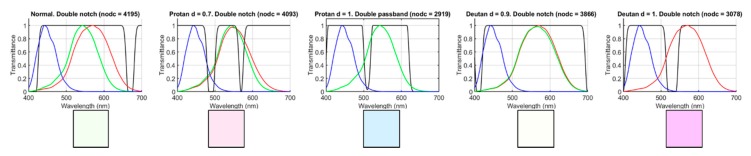
Spectral transmittances of the filters that maximize the NODC for each type of observer studied.

The NODC has been computed for the five types of observers and the 90,922 filters, using the database D1. For each observer, the filter that maximizes NODC has been chosen (see Figure 5). Table 1 shows the NODC with both the chosen filter and without a filter, the type of filter, and the relative variation of NODC (ΔNODC), computed as shown in Equation (4) where the sub-index f denotes filtered:

(4) $$\Delta NODC\left(\%\right)=\frac{NODC_{f}-NODC}{NODC}\cdot 100$$

The relative variation of NODC for dataset D1 (ΔNODC) was small in all cases, on average 1.76% higher for dichromats than for anomalous trichromats. These relative variation data were similar to those reported by Linhares et al. [18]. The fact that for simulated anomalous trichromats there was a relative decrease in NODC for dataset D2 suggests that the relative net variation was close to zero, and so the effect of the filters was of little relevance. The differences between results obtained for datasets D1 and D2 were to be expected, given the differences in the chromatic gamut spanned by the two reflectance datasets (see Figure 1 and Figure 2).

Figure 5 shows the spectral transmittances of the filters that maximize NODC for each observer, along with their LMS spectral responsivity curves. The color of the square patches below the graphs simulates approximately the appearance of each filter as seen by a normal observer. 

Figure 5 and Table 1 show that, in all cases except for the deuteranopic simulated observer, the filter that maximized NODC was a double notch filter. In any case, the double band-pass filter obtained for the deuteranopic simulated observer was very similar to a double notch. It is relevant to comment on the fact that for dichromats, the selected filters had a high transmittance in most of the wavelength range covered by the cone responsivity curves, and they included a narrow band of lower transmittance in between the two curves. For the normal and deuteranomalous subjects, the selected filters only had a low transmittance in the extreme portions of the visible range (especially in the blue region). For the protanomalous observer, the selected filter had two narrow bands of low transmittance, one in the area between the S and M and the other in the area between the L and M curves. 

For all observers, it should be noted that there were more filters that produced an increase in the NODC, even with minimal differences with respect to the selected filters. In Table 2, the percentage of filters that produced a positive relative variation in NODC is shown for each kind of filter and each simulated observer, with the type of filter that corresponds to those selected written in bold characters. 

For dichromats there was a higher relative number of filters that produce an increase in NODC, in comparison with anomalous trichromats. Neither of the two commercial filters studied produced an increase in NODC for any of the observers (see Table 3). The same can be said about the simple band-pass filters. For double band-pass filters, only the dichromats registered an increase in NODC for some of these types of filters.

### 3.2. Effects Produced by the Selected Filters

Once the filters that maximized NODC were selected for each simulated observer, the change that these filters produce on average on the color coordinates L*, a*, b* and the perceptual attributes Chroma (C*_ab_) and hue (*h_ab_*) were determined using datasets D1 and D2. Moreover, the shift caused by the filter on the color distributions of L*, a*, b* was also analyzed for both datasets. 

#### 3.2.1. Changes in Color Coordinates

In Table 4, the L*, a*, b* average shifts caused by the selected filters are shown for both datasets and each observer. The increments were computed by taking the unfiltered condition as reference.

As expected, lightness (L*) decreased when a filter was introduced since the filter partially absorbs the incident light and no lightness or chromatic adaptations were considered. The trends found for the a* and b* coordinates were similar for both datasets and each observer. This can be explained by considering that the filter shifts the color coordinates in the same direction for both datasets. For different observers, however, the shifts differed because the selected filters were different and the shifts introduced were not in the same direction.

**Figure 6:**
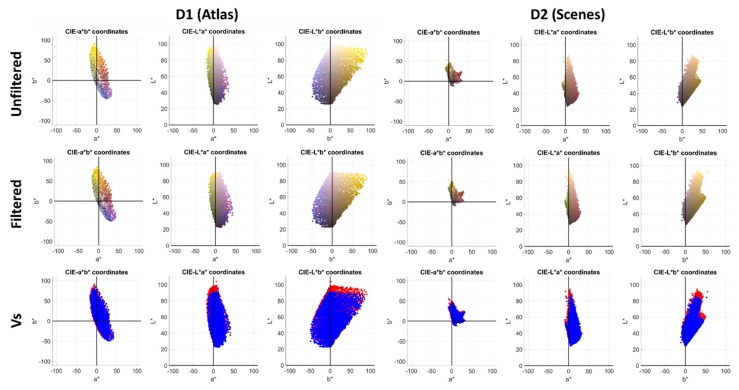
L*, a*, b* data clouds for the protanomalous simulated observer. Left: D1 dataset. Right: D2 dataset. First row: unfiltered; second row: filtered; third row: both filtered and unfiltered distributions in the same graph (red = unfiltered, blue = filtered).

Regarding C*_ab_ and h_ab_ shifts, different trends were found regarding the sign of the shift for both datasets. If the L*, a*, b* distributions were examined with and without the filter (see Figure 6), it was observed that the number of samples in each quadrant was different for each of the two datasets. Then, even when the shifts were in the same direction, the resultant changes in hue and chroma on average were different depending on the number of samples present in each quadrant.

#### 3.2.2. sRGB Renderings

The sRGB values for each sample can be obtained straightforwardly from the *XYZ* values [33]. Then, each of the scenes can be rendered for visualizing the effect produced by the selected filters. It is important to note here that the sRGB rendering presented here has not been obtained using a chromatic adaptation transformation, so the appearance of the scenes could correspond to the observers’ perception in the instant just after looking through the filter and before chromatic adaptation takes place. 

**Figure 7:**
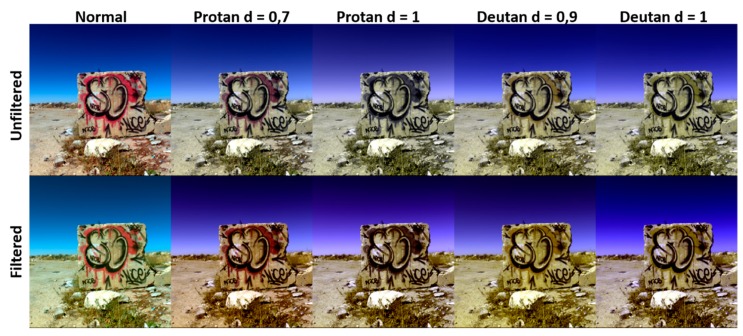
sRGB rendering of one of the scenes in dataset D2, for all simulated observers. First row: unfiltered. Second row: filtered.

The sRGB rendering for one of the scenes in dataset D2 is shown in Figure 7, for all simulated observers in both filtered and unfiltered conditions. The images have been normalized to [0,1] range to compensate for differences in lightness and allow for a comparison with a similar signal level in each case. There was a perceptible change in color for all the simulated observers, reflecting the shift in color coordinates shown in Figure 6.

**Figure 8:**
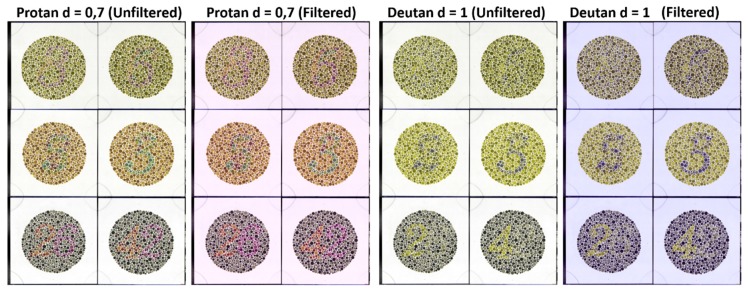
sRGB rendering of the Ishihara plates in dataset D3, for the protanomalous (left side) and deuteranopic (right side) simulated observers, for both unfiltered and filtered conditions.

In Figure 8, the six Ishihara plates belonging to dataset D3 are shown as sRGB renderings, for the protanomalous and deuteranope observers, with and without a filter. It can be observed that the effect of the filters on the appearance of the plates was not very relevant. These filters did not allow the simulated observers to pass the Ishihara test. As is shown in [10], the VINO commercial filter produced the opposite effect and allowed the simulated observers to recognize the numbers or tracks in the Ishihara plates, because this filter shifts all colors to the fourth quadrant and causes an increase in the contrast between the number and the background for CVD subjects, at the cost of substantially changing their color appearance. However, as shown in Table 3, this filter caused a decrease in the NODC for all simulated observers. 

#### 3.2.3. FM100 Results

**Figure 9:**
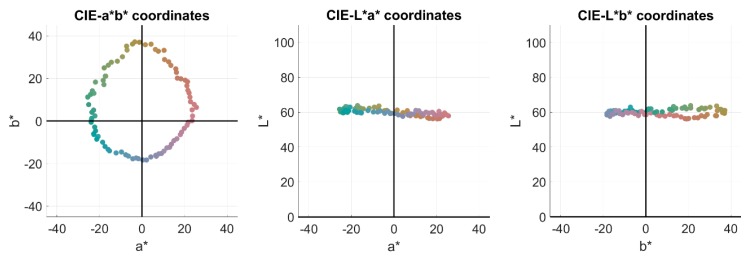
L*, a*, b* data for the FM100 samples under D65, for normal observers in unfiltered conditions.

The results of the FM100 [28] test were simulated for both filtered and unfiltered conditions using the selected filters for each observer. A normal observer should perform almost flawlessly, whilst the performance of CVD observers would depend on the severity of their condition. To simulate the test result, the spectral reflectance data of the 85 chips of the test were measured with a spectro-radiometer. Then, the L*, a*, b* values for each sample were computed using the D65 standard illuminant. The L*, a*, b* distribution for the normal observer in unfiltered conditions is shown in Figure 9.

To obtain the simulated test result, the samples were sorted according to the hue angle. Each chip was assigned a number from 1 to 85 in ascending hue order. The L*, C*_ab_ and *h_ab_* parameters have also been computed. 

**Figure 10:**
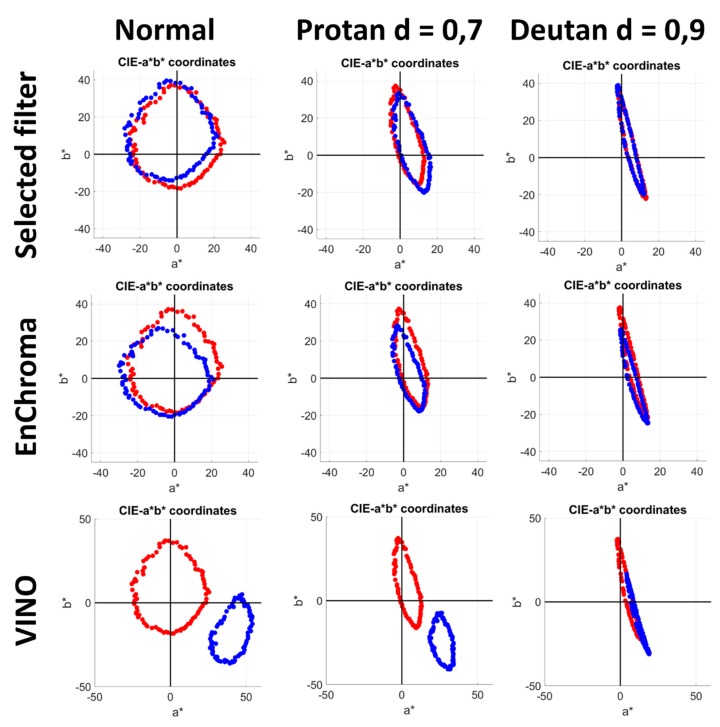
a*-b* color charts with FM100 samples plotted for normal (left), protanomalous (middle), and deuteranomalous (right) simulated observers, in the filtered (blue) and unfiltered (red) conditions. First row: selected filter; second row, EnChroma filter; third row: VINO filter.

In Figure 10, the a*-b* distributions for the three trichromat simulated subjects are shown (normal observer on the left, protanomalous in the middle, and deuteranomalous on the right), without (red) and with the selected filter for each observer (blue). The distributions for the two commercial filters EnChroma and VINO are also shown for all observers. 

The filters produced a shift in the a*, b* coordinates in all cases. This shift made it impossible for the normal observers to perform correctly when using the VINO filter. The reason for this was that the position of the center of the ring formed by the test samples in the a*-b* chart changed in the filtered condition. Hence, since the new center was not close to the origin, sorting by hue no longer produced correct results, because the reference for sorting the samples was the a* positive axis and in the filtered ring, there could be more than one sample with the same hue angle. For CVD observers, even the unfiltered sample ring was not centered in the origin, reflecting that they were unable to perform the sorting task correctly for either filtered or the unfiltered conditions. Only the normal observers with the EnChroma filter were able to perform correctly because the shift caused by the filter was not enough to disrupt the sorting process.

In Table 5 the quadratic Total Error Score (SQR), the confusion index (CI), the scatter index (SI), and Angle parameters used in the standard FM100 evaluation of results are shown for each simulated observer in both filtered and unfiltered conditions. The definition of each of these metrics can be found in [40].

The FM100 evaluation parameters for the normal observers did not change when the selected filter was introduced, so the filter did not alter the ability of the normal subject to correctly sort the FM100 samples. Regarding the CVD simulated observers, SQR values increased slightly for the deutan type observers, and they increased considerably for the protan type observers. The filter was thus worsening the performance to a higher degree for the protan type subjects. As shown in Figure 6, the effect of the selected filter for protanomalous observers was a shift towards the right in a* values. Then, some samples with negative a* values in the unfiltered condition had positive a* values in the filtered condition, with a very noticeable effect on the sorting task based on hue. Regarding the angle values, Table 5 shows that they increased for deutan subjects and decreased for protan subjects, reflecting that the distribution of responses was increasingly different for both groups. This trend also was found in a previous study using the VINO filter [10]. CI values increased for protan observers, in agreement with the trends commented above for the SQR data, while they slightly decreased for the deutan subjects. The SI values showed a trend towards decreasing or they remained the same for all subjects. The SI parameter was thus less sensitive to the effect of introducing the filter, reflecting that the randomness of the sorting was kept approximately equal when comparing the filtered and unfiltered conditions. It was not possible to analyze the statistical significance of these findings, since there was only a single instance for each simulated observer.

## 4. Conclusions

In this study, simulations were carried out to determine which spectral filter maximizes the number of discernible colors (NOCD) for normal observers, as well as four different types of red–green CVD observers (anomalous trichromats and dichromats both protan and deutan). Simulations were carried out using standard colorimetry for both normal and CVD observers. For this purpose, a set of 90,920 simulated filters (composed of single and double band-pass and notch filters) were studied, together with two measured commercial filters released as passive aids for CVD observers by the VINO and EnChroma companies.

Results on the choice of filters showed that, in all cases, the type of filter that maximized the NODC was a double notch, except for the protanope dichromats which was a double band-pass. Nonetheless, variations in NODC were small (maximum of 2.84%), and oscillated around 0% when different reflectance datasets were used. Moreover, both the EnChroma Cx-65 and VINO O2 Oxy-Iso commercial filters were shown to decrease the NODC for all studied observers and reflectance datasets. In this regard, the single band-pass filters did not yield any NODC increase either.

sRGB renderings from D2 and D3 were carried out for the five types of observers, both with and without a filter. In case of dataset D3: Ishihara, the chosen filters were shown not to include any improvement, which lead CVD observers to fail this test even when using these filters. On the other hand, results from FM100 test using these filters showed that CVD observers were unable to pass this test either, or even get close to the filterless performance of normal observers. FM100 seems to be the most reliable test for evaluating the efficacy of colored filters in color perception. Regarding the commercial filters, the EnChroma filter did not improve the performance of either the Ishihara test or the FM100 test, whilst the VINO filter helped CVD observers recognize the numbers in the Ishihara test but did not allow any observer (including normal observers) to pass the FM100 test.

These results, assuming the limitations of using standard colorimetry for CVD observers, support the hypothesis that, even though some filters may slightly increase the number of discernible colors, they can never allow CVD observers to perceive colors more similarly to normal observers. Even normal observers can stop perceiving colors correctly when using some of these filters, and they become unable to pass the FM100 test. In the future, if new personalized colorimetry frameworks are developed, especially for CVD observers, it would be very interesting to compare the results obtained to quantify the effects of these filters with the ones presented in this article.

## Figures and Tables

**Table 1:** Number of discernible colors (NODC) before and after filtering with the best-performing filter chosen for each observer and the relative increase for both dataset D1 (Atlas) and D2 (scenes).

Observer	Filter Type	NODC Filterless (D1)	NODC Filtered (D1)	ΔNODC (D1) (%)	NODC Filterless (D2)	NODC Filtered (D2)	ΔNODC (D2) (%)
Normal	Double notch	4192	4195	0.08	30,689	29,967	−2.35
Protan *d* = 0.7	Double notch	4061	4093	0.79	16,024	15,867	−0.98
Protan *d* = 1	Double band-pass	2855	2919	2.24	2634	2685	1.94
Deutan *d* = 0.9	Double notch	3836	3866	0.78	7690	7686	−0.05
Deutan *d* = 1	Double notch	2993	3078	2.84	3176	3238	1.95

**Table 2:** Global percentage of filters of each type that produced a positive relative variation in NODC for each observer. The data for the filters of the same type as the one selected are shown in bold numbers.

Observer	Global (%)	Notch 1 (%)	Pass 1 (%)	Notch 2 (%)	Pass 2 (%)
Normal	0.01	0	0	**0.02**	0
Protan *d* = 0.7	0.79	5.18	0	**0.43**	0
Protan *d* = 1	3.04	18.45	0	4.47	**0.13**
Deutan *d* = 0.9	0.23	4.85	0	**0.43**	0
Deutan *d* = 1	2.39	18.45	0	**4.47**	0.22

**Table 3:** NODC before and after filtering with the EnChroma and VINO filters, and the relative increase for dataset D1 (Atlas).

Observer	NODC Unfiltered	NODC with EnChroma	ΔNODC EnChroma (%)	NODC with VINO	ΔNODC VINO (%)
Normal	4192	4168	−0.57	3934	−6.15
Protan *d* = 0.7	4061	3980	−1.99	3735	−8.03
Protan *d* = 1	2855	2578	−9.70	1549	−45.74
Deutan *d* = 0.9	3836	3680	−4.07	2849	−25.73
Deutan *d* = 1	2993	2676	−10.59	2323	−22.39

**Table 4:** Mean (standard deviation) of shifts in color coordinates when the selected best-performing filters were used by each simulated observer, and for both dataset D1 (Atlas) and D2 (scenes).

	D1					D2				
Observer	ΔL*	Δa*	Δb*	ΔC*	Δ*h_ab_*	ΔL*	Δa*	Δb*	ΔC*	Δ*h_ab_*
Normal	−0.23	−3.55	3.25	0.55	−0.13	−0.20	−2.52	2.11	1.40	7.96
	(0.12)	(1.06)	(0.95)	(3.47)	(28.04)	(0.08)	(0.36)	(0.28)	(1.15)	(19.02)
Protan *d* = 0.7	−5.36	2.82	−3.55	0.72	−11.10	−4.39	2.20	−2.65	−1.25	−15.33
	(1.27)	(1)	(1.31)	(3.63)	(18.21)	(0.86)	(0.41)	(0.34)	(1.52)	(8.59)
Protan *d* = 1	−3.29	1.13	−4.29	0.32	13.84	−2.48	0.63	−3.11	−2.24	−10.87
	(1.16)	(0.71)	(1.78)	(3.75)	(14.00)	(0.55)	(0.21)	(0.75)	(1.57)	(9.70)
Deutan *d* = 0.9	−0.09	−0.65	1.88	−0.01	5.63	−0.06	−0.36	1.24	0.94	3.6
	(0.04)	(0.29)	(0.5)	(1.69)	(6.61)	(0.01)	(0.08)	(0.12)	(0.61)	(3.48)
Deutan *d* = 1	−4.13	1.69	−5.97	1.11	−14.55	−2.98	0.89	−4.24	−2.85	−14.77
	(1.63)	(1.13)	(2.52)	(5.63)	(16.86)	(0.6)	(0.28)	(0.89)	(2.37)	(14.31)

**Table 5:** Quadratic Total Error Score (SQR), Angle, C-Index, and S-Index computed for each simulated observer in the filtered (with the best-performing filter) and unfiltered conditions.

Observer	SQR (unfilt)	SQR (filt)	Angle (unfilt)	Angle (filt)	CI (unfilt)	CI (filt)	SI (unfilt)	SI (filt)
Normal	2	2	49.58	49.58	1.05	1.05	1.33	1.33
Protan *d* = 0.7	6	12.96	61.47	54.14	1.28	2.4	1.46	1.3
Protan *d* = 1	17.55	19.80	43.17	42.52	5.89	5.03	2.07	1.85
Deutan *d* = 0.9	15.23	15.62	43.3	46.32	4.61	4.47	1.64	1.63
Deutan *d* = 1	16.97	17.32	48.72	56.17	3.95	3.7	1.45	1.27

## References

[1] Birch J. (2012). Worldwide prevalence of red-green color deficiency. JOSA A.

[2] Simunovic M.P. (2010). Colour vision deficiency. Eye.

[3] Went L.N., Pronk N. (1985). The genetics of tritan disturbances. Hum. Genet..

[4] Cole B.L. (2004). The handicap of abnormal colour vision. Clin. Exp. Optom..

[5] Neitz M., Neitz J. (2014). Curing color blindness—mice and nonhuman primates. Cold Spring Harb. Perspect. Med..

[6] Popleteev A., Louveton N., McCall R. (2015). Colorizer: Smart glasses aid for the colorblind. WearSys.

[7] Simon-Liedtke J.T., Farup I. Multiscale Daltonization in the Gradient Domain. Proceedings of the Color and Imaging Conference.

[8] (2020). EnChroma, EnChroma Inc. https://enchroma.com/.

[9] VINO. https://www.vino.vi/collections/color-blind-glasses.

[10] Martínez-Domingo M.A., Gómez-Robledo L., Valero E.M., Huertas R., Hernández-Andrés J., Ezpeleta S., Hita E. (2019). Assessment of VINO filters for correcting red-green Color Vision Deficiency. Opt. Express.

[11] Gómez-Robledo L., Valero E.M., Huertas R., Martínez-Domingo M.A., Hernández-Andrés J. (2018). Do EnChroma glasses improve color vision for colorblind subjects?. Opt. Express.

[12] Almutairi N., Kundart J., Muthuramalingam N., Hayes J., Citek K., Aljohani S. Assessment of Enchroma Filter for Correcting Color Vision Deficiency. https://commons.pacificu.edu/work/501bb1bb-1502-475e-aa5e-d8f2d7abde1f.

[13] Mastey R., Patterson E.J., Summerfelt P., Luther J., Neitz J., Neitz M., Carroll J. (2016). Effect of “color-correcting glasses” on chromatic discrimination in subjects with congenital color vision deficiency. Investig. Ophthalmol. Vis. Sci..

[14] Patterson E.J. Glasses for the colorblind: Their effect on chromatic discrimination in subjects with congenital red-green color vision deficiency. Proceedings of the International Conference on Computer Vision Systems (ICVS).

[15] Lucassen M., Alferdinck J. Dynamic simulation of color blindness for studying color vision requirements in practice. Proceedings of the Conference on Colour in Graphics, Imaging, and Vision.

[16] Machado G.M., Oliveira M.M., Fernandes L.A.F. (2009). A physiologically-based model for simulation of color vision deficiency. IEEE Trans. Vis. Comput. Graph..

[17] Wachtler T., Dohrmann U., Hertel R. (2004). Modeling color percepts of dichromats. Vis. Res..

[18] Linhares J.M.M., Pinto P.D., Nascimento S.M.C. (2008). The number of discernible colors perceived by dichromats in natural scenes and the effects of colored lenses. Vis. Neurosci..

[19] Brettel H., Viénot F., Mollon J.D. (1997). Computerized simulation of color appearance for dichromats. JOSA A.

[20] Moreland J.D., Westland S., Cheung V., Dain S.J. (2010). Quantitative assessment of commercial filter ‘aids’ for red-green colour defectives. Ophthalmic Physiol. Opt..

[21] DeMarco P., Pokorny J., Smith V.C. (1992). Full-spectrum cone sensitivity functions for X-chromosome-linked anomalous trichromats. JOSA A.

[22] Marín-Franch I., Foster D.H. (2010). Number of perceptually distinct surface colors in natural scenes. J. Vis..

[23] Pastilha R.C., Linhares J.M.M., Gomes A.E., Santos J.L.A., Almeida V.M.N., Nascimento S.M.C. (2019). The colors of natural scenes benefit dichromats. Vis. Res..

[24] Masuda O., Nascimento S.M.C. (2012). Lighting spectrum to maximize colorfulness. Opt. Lett..

[25] Schrödinger E. (1920). Theorie der Pigmente von grösster Leuchtkraft. Ann. Phys..

[26] Thornton W.A. (1972). Color-discrimination index. JOSA.

[27] Ishihara S. (1987). Test for Colour-Blindness.

[28] Rite X. (2006). FM 100 Hue Color Vision Test. 100 Hue Test Scoring Tool, Version 3.0. Munsell.

[29] Joint I.S.O. (1999). CIE Standard Illuminants for Colorimetry. ISO.

[30] Nickerson D. (1940). History of the Munsell color system and its scientific application. JOSA.

[31] Hård A., Sivik L. (1981). NCS—Natural Color System: A Swedish standard for coloer notation. Color Res. Appl..

[32] Sutton T., Whelan B.M. (2017). The Complete Color Harmony, Pantone Edition: Expert Color Information for Professional Color Results.

[33] Stokes M., Anderson M., Chandrasekar S., Motta R. A Standard Default Color Space for the Internet-Srgb. https://www.w3.org/Graphics/Color/sRGB.

[34] Arad B., Ben-Shahar O. Sparse recovery of hyperspectral signal from natural RGB images. Proceedings of the European Conference on Computer Vision.

[35] Clark J.H. (1924). The Ishihara test for color blindness. Am. J. Physiol. Opt..

[36] (2019). Resonon, Resonon Inc. https://resonon.com/Pika-L.

[37] Davidoff C., Neitz M., Neitz J. (2016). Genetic testing as a new standard for clinical diagnosis of color vision deficiencies. Transl. Vis. Sci. Technol..

[38] Fairman H.S., Brill M.H., Hemmendinger H. (1997). How the CIE 1931 color-matching functions were derived from Wright-Guild data. Color Res. Appl..

[39] Mokrzycki W.S., Tatol M. (2011). Colour difference∆ E-A survey. Mach. Graph. Vis..

[40] Vingrys A.J., King-Smith P.E. (1988). A quantitative scoring technique for panel tests of color vision. Investig. Ophthalmol. Vis. Sci..

